# Transcriptome analysis gene expression in the liver of *Coilia nasus* during the stress response

**DOI:** 10.1186/1471-2164-15-558

**Published:** 2014-07-04

**Authors:** Fukuan Du, Gangchun Xu, Zhijuan Nie, Pao Xu, Ruobo Gu

**Affiliations:** Key Laboratory of Freshwater Fisheries and Germplasm Resources Utilization, Ministry of Agriculture, Freshwater Fisheries Research Center, Chinese Academy of Fishery Sciences, No. 9 Shanshui East Road, Wuxi, Jiangsu Province China

**Keywords:** Gene expression, *Coilia nasus*, Stress, Liver injury, Apoptosis

## Abstract

**Background:**

The estuarine tapertail anchovy (*Coilia nasus*) is widely distributed in the Yangtze River, the coastal waters of China, Korea, and the Ariake Sound of Japan. It is a commercially important species owing to its nutritional value and delicate flavor. However, *Coilia nasus* is strongly responsive to stress, this often results in death, which causes huge losses. In this study, we used next-generation sequencing technologies to study changes in gene expression in response to loading stress and the mechanism of death caused by loading stress in *Coilia nasus*.

**Results:**

Using next-generation RNA-seq technologies on an Illumina HiSeq 2000 platform, we assembled a de novo transcriptome and tested for differential expression in response to stress. A total of 65,129 unigenes were generated, the mean unigene size and N50 were 607 bp and 813 bp, respectively. Of the assembled unigenes, we identified 2,990 genes that were significantly up-regulated, while 3,416 genes were significantly down-regulated in response to loading stress. Pathway enrichment analysis based on loading stress-responsive unigenes identified significantly stress related pathways. “Metabolism” and “immunity” were the two most frequently represented categories. In the “metabolism” category, “glucose metabolism” and “lipid metabolism” were major subclasses. The transcriptional expression of rate-limiting enzymes in “glucose metabolism” and “lipid metabolism” was detected by RT-qPCR, all were significantly increased after stress. Apoptosis associated proteins tumor necrosis factor alpha (TNF-α), caspase 9, cytochrome c and caspase 3 in the stress group were significantly elevated, moreover, liver injury indicators (Alanine aminotransferase, ALT, and aspartate transaminase, AST) were also significantly elevated, which indicates that loading stress induced liver injury.

**Conclusion:**

This study provided abundant unigenes that could contribute greatly to the discovery of novel genes in fish. The alterations in predicted gene expression patterns reflected possible responses to stress. Loading stress may induce liver injury through the mitochondrial apoptosis pathway, which was activated by TNF-α. Taken together, our data not only provide information that will aid the identification of novel genes from fish, but also shed new light on the understanding of mechanisms by which physical stressors cause death in fish.

**Electronic supplementary material:**

The online version of this article (doi:10.1186/1471-2164-15-558) contains supplementary material, which is available to authorized users.

## Background

Stress exists in all aspects of aquaculture, daily management such as netting, loading and transporting can lead to a stress response. Stress may result in inhibition of growth, reproductive failure, and reduced resistance to pathogens [1–5]. The estuarine tapertail anchovy (*Coilia nasus*, junior synonym *C. ectenes*) is widely distributed in the Yangtze River, the coastal waters of China, Korea, and the Ariake Sound of Japan [6]. It is a commercially important species owing to its nutritional value and delicate flavor. However, *Coilia nasus* is strongly responsive to stress, this often results in death, which causes huge losses. Currently, little is known of the mechanism by which loading stress causes death in fish. In this study, we used next-generation sequencing technologies to study gene expression changes in response to loading stress and the mechanism of death caused by loading stress in *Coilia nasus*.

Recently, several species-specific cDNA microarrays have been developed for teleosts, and they are being used increasingly to reveal global gene expression patterns in response to stressor exposure and/or hormone treatment [7–10]. These studies in fish have revealed that the majority of changes, inspite of the use of different arrays and different types of acute stressor, can be broadly categorized functionally into genes encoding proteins involved in metabolism, immune function and reproduction. Although these studies have identified several genes that were previously not known to be stress responsive, the significance of these observed transcript changes to overall stress adaptation is far from clear.

While the previous studies that used various microarray platforms in fish were limited by large representation of expressed sequence tags (EST), which made gene-specific interpretation of the data difficult, the advent of next-generation sequencing methods, including pyrosequencing, has effectively overcome this limitation [11, 12]. Rapid progress in next-generation sequencing technologies has allowed large-scale efficient and economical production of ESTs. Transcriptome sequencing facilitates functional genomic studies, including global gene expression, novel gene discovery, and assembly of full-length genes [13–16]. To date, available molecular information on *Coilia nasus* remains sparse, which limits research on the mechanism by which loading stress causes death. The powerful new technologies provide a new opportunity for studies of species without genome reference databases, and non-model organisms.

## Methods

### Experimental animals

*Coilia nasus* (average weight, 9.6 g) were adapted to the conditions in a 7.0 × 5.0 × 1.0 m^3^ aquarium with a water temperature of 24.5 ± 1.0°C, pH 7.2, and dissolved oxygen concentration of 9.2 ± 0.5 mg O_2_/L dechlorinated and aerated water. The fish were fed twice daily, at 7:00 AM and 5:00 PM. At the onset of the experiments, all fish appeared healthy.

### Stressing experiment

In March 2012, three 7.0 × 5.0 × 1.0 m^3^ ponds were stocked with 120 juvenile *Coilia nasus* each. The fish were acclimated to the ponds for approximately 14 months before the experiment, at which time the fish were 15 months old. Excess fish were stocked in the ponds in order to ensure subsequent access to the intended numbers. In detail, *Coilia nasus* prefer to swim towards lighted areas in dark situations. To take advantage of this phototaxis, a sifter was put into the water and lit, following which the fish moved into the sifter. The fish were euthanized immediately with 70 mg/L buffered tricaine methanesulfonate (MS-222). Using this method, five fish were removed from each pond, these 15 fish were the non-stressed controls and were processed immediately (see tissue sampling below). Subsequently, another five fish were netted from each pond and loaded into 75 × 55 × 33 cm3 tanks. After 0.5 h, the fish were euthanized as described above. Compared with the stressed fish, they did not experience the netting and handling used for the control fish. The mean length was 136.98 mm ± 9.26 SEM and mass was 8.86 g ± 1.76 SEM for all fish (*n* = 30) sampled in this experiment.

### Tissue sampling

During the experiments, euthanized fish were submerged immediately in crushed ice to retard degradation of RNA. Blood was collected into ammonium-heparinized capillary tubes after severance of the caudal fin. All fish appeared healthy during dissection and their livers were removed and placed in liquid nitrogen. Plasma was separated by centrifugation. Plasma and liver samples were stored at -80°C until later analysis.

Animal welfare and experimental procedures were carried out in accordance with the Guide for the Care and Use of Laboratory Animals (Ministry of Science and Technology of China, 2006), and were approved by the animal ethics committee of the Chinese Academy of Fishery Sciences.

### Cortisol and glucose

Blood glucose measurement was performed by glucose-oxidase-superoxide enzyme endpoint colorimetry, the test kit was obtained from Shanghai Biological Product Research Institute, Ministry of Public Health, China. Cortisol was measured by radioimmunoassay in accordance with the method described by Pickering and Pottinger [17], the test kit was purchased from Beijing Beifang Biotech Research Institute, China. Plasma samples were measured using a Beckman Cx-4 spectrophotometer (Beckman Coulter, Fullerton, CA, USA). Student’s t test was used to analyze differences among all treatments (P < 0.05).

### RNA sequencing, assembly and annotation

Transcriptome sequencing was carried out on an Illumina HiSeq 2000 platform that generated approximately 100-bp paired-end (PE) raw reads (BGI, Shenzhen, China). After removing adaptor sequences, ambiguous ‘N’ nucleotides (with the ratio of ‘N’ greater than 5%) and low quality sequences (with quality score less than 10), the remaining clean reads were assembled using trinity software [18] as described for de novo transcriptome assembly without a reference genome. For homology annotation, non-redundant sequences were subjected to public databases, including NCBI (http://www.ncbi.nlm.nih.gov/) non-redundant protein (Nr) and non-redundant nucleotide (Nt), Swiss-Prot (http://www.ebi.ac.uk/uniprot/), Gene Ontology (GO, http://www.geneontology.org/), Clusters of Orthologous Groups (COG, http://www.ncbi.nlm.nih.gov/COG/) and the Kyoto Encyclopedia of Genes and Genomes (KEGG, http://www.genome.jp/kegg/). If the results from different databases conflicted, a priority order of alignments from Nr, Nt, KEGG, Swiss-Prot, GO and COG databases was followed. Comparison with the Nr, Nt and Swiss-Prot databases was carried out using the BlastX algorithm with an E-value cut-off of 0.00001; GO terms at the 2nd level were used to perform GO annotation; COG and KEGG classification were performed using BlastX with an E-value cut-off of 0.00001.

### Analysis of differentially expressed genes

To analyze stress-responsive, differentially expressed genes in *Coilia nasus*, the number of reads for each of the contigs from the two samples was converted to reads per kilo base per million (RPKM) [19]. Following this, the MA-plot-based method with Random Sampling model (MARS) in the DEGseq package was used to calculate the expression abundance of each contig among the analyzed samples. We used an FDR (false discovery rate) to determine the P-value threshold. An FDR < 0.001 was considered to indicate significant expression abundance.

Pathway enrichment analysis identifies significantly enriched metabolic pathways or signal transduction pathways in differentially expressed genes by comparing them with the whole genome background. Bonferroni adjustments [20] were used to estimate levels of significance. After correction for multiple testing, we chose pathways with a Q-value ≤ 0.05 to represent those significantly enriched in differentially expressed genes. The Q-value is defined to be the FDR analog of the P-value. The Q-value of an individual hypothesis test is the minimum FDR at which the test maybe reported as significant.

### Gene expression validation

Genes identified in this transcriptome sequencing analysis were validated and quantified by real-time PCR (RT-qPCR). The primers (Additional file 1: Table S1) were designed according to Illumina sequencing data with Primer Premier 5. The prepared total RNA used in RT-PCR analysis was isolated from the same sample as that used for Illumina sequencing. The RT-qPCR was performed on the ABI 7500 real-time PCR system (ABI, USA) using 2× SYBR green real-time PCR mix (Takara, Japan). The PCR amplification was performed in triplicate, using the following cycling parameters: 94°C for 2 min, followed by 40 cycles of 15 s at 94°C, 15 s at 60°C, and 34 s at 72°C. All samples were analyzed in triplicate and the expression of target genes was calculated as relative fold values using the 2^-△△CT^ method.

### Analysis of liver lipid peroxides, tumor necrosis factor alpha and apoptosis-associated proteins

Hepatocyte damage following stress was assessed by measuring alanine aminotransferase (ALT) and aspartate transaminase (AST) activities in plasma, using corresponding detection kits (Jusbio, Shanghai, China) according to the manufacturer**’**s instructions. TNF-α, cytochrome c, caspase-9, and caspase-3 in hepatic tissue were analyzed using an enzyme-linked immunosorbent assay kit (Zhaorui, Shanghai, China), as described by the manufacturer. Lipid peroxides (LPO) were detected by colorimetric methods, using a kit (Jiancheng, Nanjing, China) according to the manufacturer’s instructions.

### Statistical analysis

Student’s *t* test or ANOVA, where appropriate, were used to identify significant differences among the treatments at p = 0.05. All data in this study were expressed as mean ± standard (S.D.).

## Results

### Plasma cortisol and glucose

**Figure 1:**
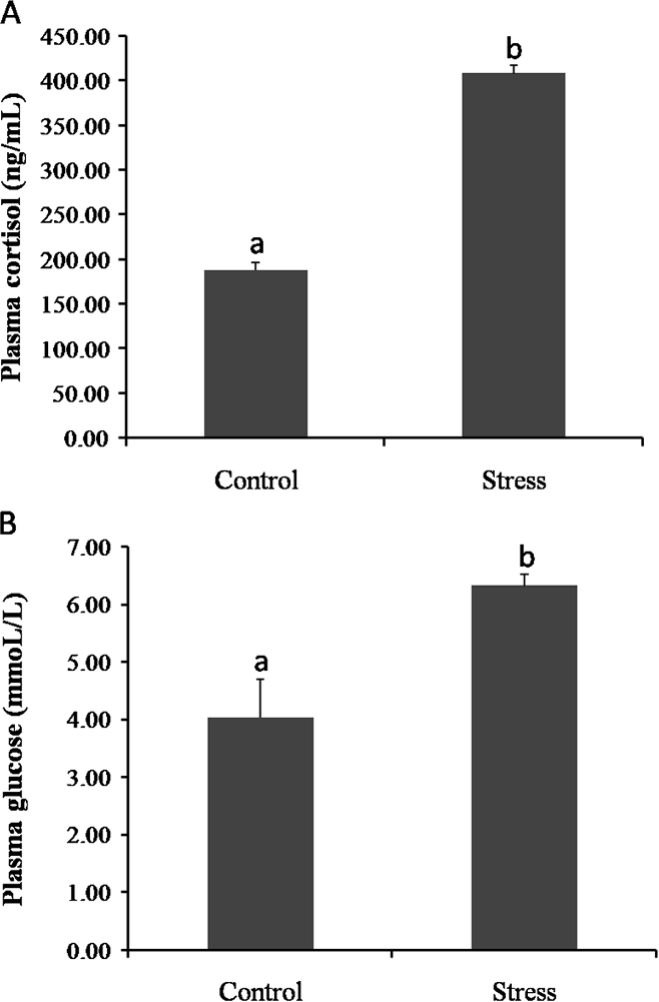
**Plasma cortisol and glucose concentrations in control and loading stress groups.** Bars represent the mean for replicates. Values with different letters are significantly different at P < 0.05. **(A)**. Plasma cortisol concentrations for control and stressed groups (*n* = 15). **(B)**. Plasma glucose levels for the two groups.

Plasma cortisol was significantly elevated (P < 0.01) in the stressed fish (407.72 ng/mL ± 9.50 SEM, *n* = 15) compared with the control fish (187.76 ng/mL ± 9.07, *n* = 15) (Figure 1A). There were no significant differences between control replicates (P > 0.05).

Plasma glucose concentrations (Figure 1B) in the stressed group (6.31 mmol/L ± 0.20, *n* = 15) were statistically higher than in the controls (4.03 mmol/L ± 0.66, P < 0.05, *n* = 15).

### Generation and de novo assembly of Coilia nasus transcriptome data

In this study, we performed transcriptome sequencing of two libraries from liver samples in *Coilia nasus* via an Illumina HiSeq 2000 platform sequencer: 64.8 and 67.8 million reads were obtained from the two libraries. After removing low-quality reads, short reads and reads belonging to mitochondria, a total of 111,053,176 clean reads corresponding to mRNAs were obtained, these reads covered a total of 9,994,785,840 bases (Table 1).

**Table 1:** **Summary of sequence data generated for the** ***Coilia nasus*** **transcriptome, and quality filtering**

Sample	Total reads	Clean reads	Clean nucleotides (nt)	Q20 percentage (%)
**Control**	64,827,364	55,526,588	4,997,392,920	98.20
**Stress**	67,782,560	55,526,588	4,997,392,920	98.28
**Total**	132,609,924	111,053,176	9,994,785,840	98.24

**Figure 2:**
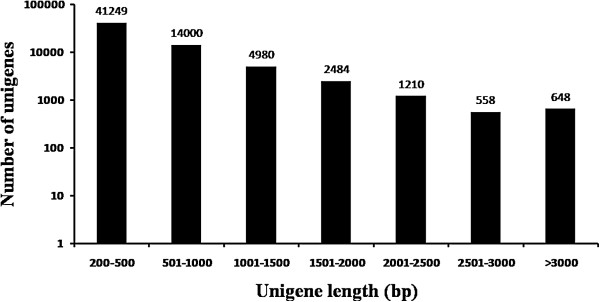
**Length distribution of unigenes.**

Using the Trinity assembly program, we generated a total of 65,129 unigenes (Table 2). The length distribution of unigenes larger than 200 bp is shown in Figure 2. The mean unigene size and N50 were 607 bp and 813 bp, respectively. About half of the unigenes (30,582; 47.0%) were ≥ 500 bp and 648 unigenes were > 3,000 bp in length. The largest unigene was 10,911 bp in length (Table 2).

**Table 2:** **Assembly statistics of reads**

Parameter	Numbers
**Number of Unigene**	65,129
**Total bases of Unigene (bp)**	39,474,010
**Unigene mean lengths (bp)**	606
**Number of Unigene ≥500 bp**	30,582
**N50**	835
**Max length (bp)**	10,911

### Functional annotation and pathway assignment

**Figure 3:**
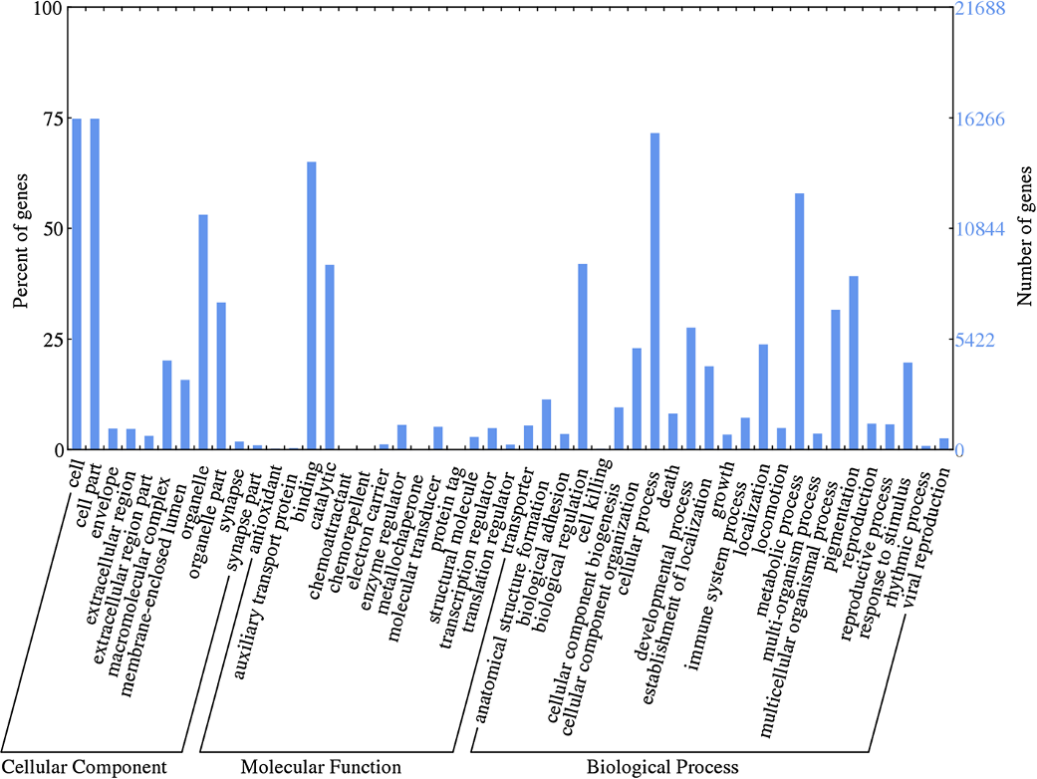
**GO categorization of non-redundant unigenes.**

According to Gene Ontology (GO), an internationally standardized gene functional classification system, 21,688 non-redundant unigenes were classified into three major functional categories (biological process, cellular component and molecular function) and 49 subcategories (Table 3, Figure 3). In the category of cellular components, “cell part” (15,015, 69.2%) were the most commonly represented, followed by “organelle” (11,679, 53.9%) and “organelle part” (7,270, 33.5%). Among the molecular function terms, a significant proportion of clusters were assigned to “binding” (14,201, 65.5%) and “catalytic activity” (9,099, 50.0%). Of sequences categorized as biological processes, dominant subcategories were “cellular process” (15,851,73.1%) and “metabolic process” (13,020, 60.0%). However, within each of the three categories, few genes were assigned to the subcategories of “growth”, “cell junction” and “receptor regulator activity”.

**Table 3:** **Blast analysis of non-redundant unigenes against public databases**

Datebase	Number of annotated unigenes	Percentage of annoted unigenes (%)
**Nr**	33,723	51.8
**Nt**	31,224	47.9
**Swiss-prot**	30,476	46.8
**KEGG**	25,188	38.7
**GO**	21,688	33.3
**COG**	10,631	16.3

**Figure 4:**
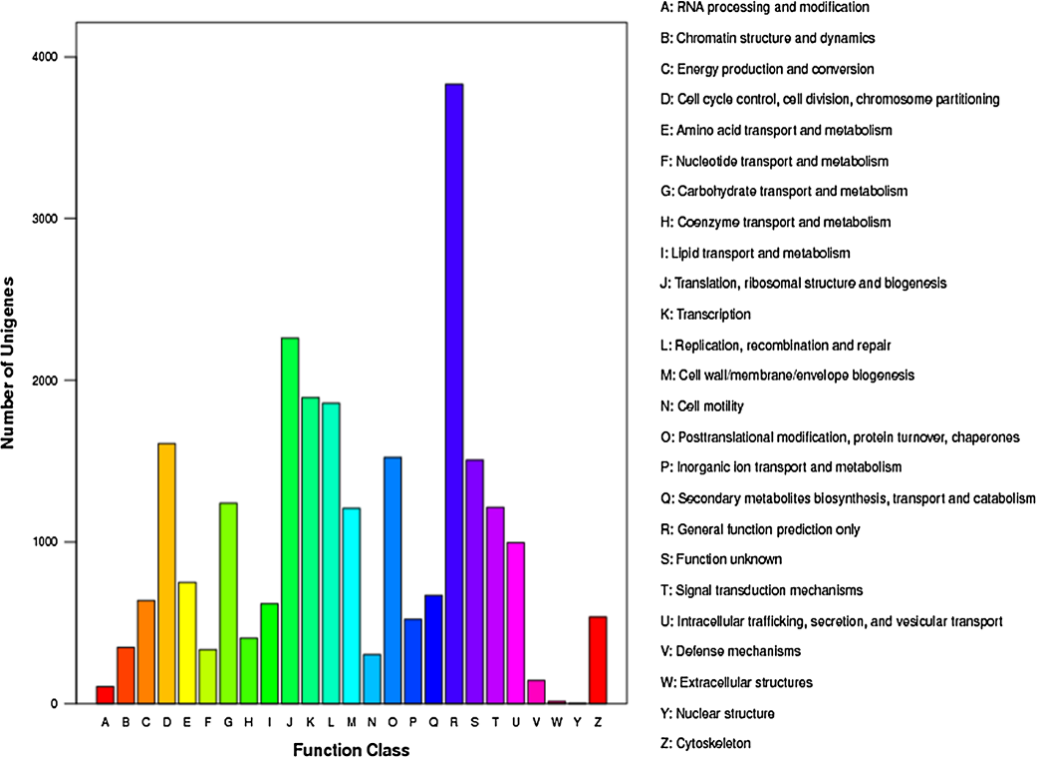
**COG annotation of putative proteins.**

To classify orthologous gene products, 10,631 (16.3%) non-redundant unigenes (Table 3) were subdivided into 25 COG classifications. Among them, the cluster of “general function prediction only” (3,830, 36.0%) represented the largest group, followed by “translation, ribosomal structure and biogenesis” (2,260, 21.3%), “transcription” (1,893, 17.8%), “replication, recombination and repair” (1,858, 17.5%), “cell cycle control, cell division, chromosome partitioning” (1,608, 15.1%), “post-translational modification, protein turnover, chaperon” (1,522, 14.3%) and “function unknown” (1,506, 14.2%), “nuclear structure” (4, 0.03%) was the smallest group (Figure 4).

The Kyoto Encyclopedia of Genes and Genomes (KEGG) pathway analysis revealed that diverse pathways were represented in our transcriptome dataset: 25,188 unigenes (Additional file 2: Table S2) were assigned to 259 specific pathways. Among them, “metabolism pathways”, “regulation of actin cytoskeleton”, “pathways in cancer”, “focal adhesion”, and “endocytosis” were the five most frequently represented pathways (Additional file 2: Table S2). Some important pathways involved in signal transduction were also identified, including “MAPK signaling pathway”, “calcium signaling pathway” and “Jak-STAT signal pathway” (Additional file 2: Table S2).

### Loading stress-responsive unigenes in Coilia nasus

**Figure 5:**
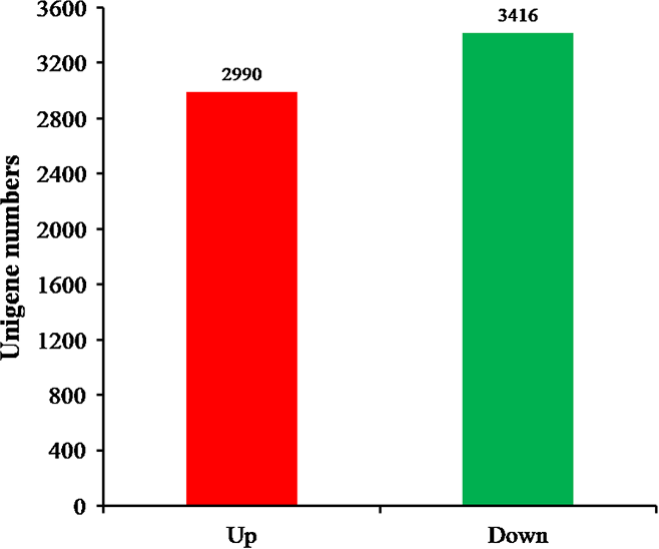
**Genes differentially expressed following stress.**

Unigene expression was estimated by the FPKM method, and differentially expressed genes were identified by referenceto Audic [21]. We found that 2,990 genes were significantly up-regulated, while 3,416 genes were significantly down-regulated in response to loading stress (Figure 5). The up-regulated and down-regulated genes are listed in Additional file 3: Table S3. The indicated genes included metabolic genes, enzymes, and other immune-related genes, such as the GTPase gene, threonine-protein kinase and MHC class I heavy chain gene. These genes showed different expression patterns after stress, which implies that they may play an important role in physiological processes associated with stress.

Pathway enrichment analysis based on loading stress-responsive unigenes identified significantly stress-related pathways. Among these pathways, “metabolism” and “immunity” were the two most commonly represented categories. In the “metabolism” category, “glucose metabolism” and “lipid metabolism” were major subclasses, and included “glycolysis/gluconeogenesis”, “starch and sucrose metabolism”, “glycerophospholipid metabolism” and “fat digestion and absorption” pathways. In the “immunity” category, “phagosome”, “HTLV-I infection” and “amoebiasis” were the most frequently represented pathways (Table 4).

**Table 4:** **KEGG pathways of differentially expressed genes**

Category	Pathway	Pathway ID	Gene number	Q-value
**Carbohydrate metabolism**	Glycolysis/Gluconeogenesis	ko00010	38	2.09E-03
	Starch and sucrose metabolism	ko00500	32	2.67E-03
	Pyruvate metabolism	ko00620	32	2.89E-03
	Type I diabetes mellitus	ko04940	31	1.39E-08
	Glyoxylate and dicarboxylate metabolism	ko00630	24	1.34E-04
	Butanoate metabolism	ko00650	17	4.16E-03
	D-Glutamine and D-glutamate metabolism	ko00471	5	2.12E-02
**Lipid metabolism**	Glycerophospholipid metabolism	ko00564	36	4.43E-02
	Fat digestion and absorption	ko04975	28	3.61E-03
	Steroid hormone biosynthesis	ko00140	24	3.40E-02
	Steroid biosynthesis	ko00100	23	1.22E-12
	Fatty acid biosynthesis	ko00061	12	9.34E-04
	Ether lipid metabolism	ko00565	16	3.56E-02
**Other metabolism**	Protein processing in endoplasmic reticulum	ko04141	92	3.53E-03
	Protein digestion and absorption	ko04974	60	1.53E-04
	Metabolic pathways	ko01100	448	2.46E-09
	Glutathione metabolism	ko00480	26	2.67E-03
	Caffeine metabolism	ko00232	8	3.56E-03
	PPAR signaling pathway	ko03320	64	2.52E-06
	Arachidonic acid metabolism	ko00590	38	6.51E-04
	Vitamin digestion and absorption	ko04977	24	1.34E-03
	Propanoate metabolism	ko00640	21	1.24E-02
	Terpenoid backbone biosynthesis	ko00900	16	5.07E-06
	alpha-Linolenic acid metabolism	ko00592	14	7.18E-03
	Valine, leucine and isoleucine biosynthesis	ko00290	8	2.67E-03
**Immunity**	Phagosome	ko04145	114	1.09E-08
	HTLV-I infection	ko05166	107	2.67E-02
	Amoebiasis	ko05146	104	2.07E-02
	Epstein-Barr virus infection	ko05169	100	2.42E-02
	Herpes simplex infection	ko05168	100	3.40E-02
	Tuberculosis	ko05152	89	7.47E-03
	Viral myocarditis	ko05416	73	2.46E-03
	Antigen processing and presentation	ko04612	72	1.17E-13
	Systemic lupus erythematosus	ko05322	68	2.59E-09
	Pertussis	ko05133	63	1.38E-02
	Complement and coagulation cascades	ko04610	60	3.61E-03
	Natural killer cell mediated cytotoxicity	ko04650	59	1.50E-03
	Staphylococcus aureus infection	ko05150	54	4.71E-04
	Rheumatoid arthritis	ko05323	51	8.59E-06
	Legionellosis	ko05134	49	2.67E-03
	Leishmaniasis	ko05140	48	2.84E-04
	Chagas disease (American trypanosomiasis)	ko05142	48	4.65E-02
	Autoimmune thyroid disease	ko05320	41	3.43E-11
	Allograft rejection	ko05330	40	3.43E-11
	Prion diseases	ko05020	40	2.67E-02
	Graft-versus-host disease	ko05332	27	5.09E-07
	Primary immunodeficiency	ko05340	27	3.53E-03
	Intestinal immune network for IgA production	ko04672	24	2.32E-04
	African trypanosomiasis	ko05143	23	1.34E-03
	Asthma	ko05310	22	8.22E-05
**Others**	Spliceosome	ko03040	113	3.29E-02
	Insulin signaling pathway	ko04910	91	1.34E-04
	Alzheimer’s disease	ko05010	65	3.57E-02
	Pancreatic secretion	ko04972	56	1.05E-05
	Ribosome	ko03010	55	1.72E-06
	Calcium signaling pathway	ko04020	55	1.38E-02
	Adipocytokine signaling pathway	ko04920	45	1.09E-02
	Gastric acid secretion	ko04971	42	1.18E-03
	Circadian rhythm - mammal	ko04710	31	1.28E-06
	DNA replication	ko03030	27	5.09E-07
	Olfactory transduction	ko04740	23	2.95E-02
	Phototransduction - fly	ko04745	19	3.40E-02
	Proximal tubule bicarbonate reclamation	ko04964	18	3.48E-03
	Mismatch repair	ko03430	16	8.76E-04
	Circadian rhythm - fly	ko04711	15	4.32E-03
	Protein export	ko03060	10	3.21E-02

### Loading stress-induced liver injury

**Figure 6:**
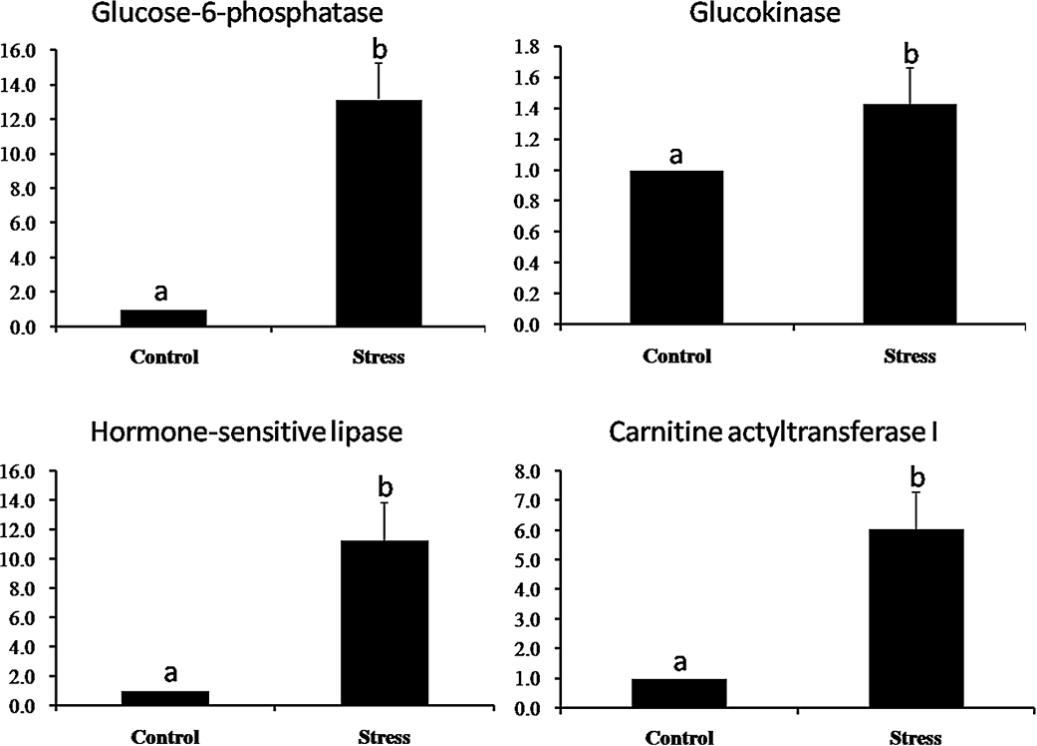
**Glucose-6-phosphatase, glucokinase, hormone-sensitive lipase and carnitine actyltransferase I mRNA expression levels in liver in response to stress.** The vertical axis shows the relative gene expression levels (means ± SD), bars represent the mean for each replicate ± SD. Replicates or treatments with different letters are significantly different at P < 0.05.

Glucose-6-phosphataseand glucokinase are rate-limiting enzymes in gluconeogenesis and glycolysis. Liver glucose-6-phosphatase and glucokinase revealed 13.2- and 1.4-fold increased expression after stressing, respectively (Figure 6; P < 0.05). Hormone-sensitive lipase and carnitine actyltransferase I are rate-limiting enzymes for adipokinetic action and fatty acid beta-oxidation. The RT-qPCR results also revealed 11.3- and 6.0-fold increased expression for liver hormone-sensitive lipase and carnitine actyltransferase I in stressed *Coilia nasus* (Figure 6; P < 0.05).

Lipid peroxides (LPO) are produced in the fatty acid beta-oxidation process, and are cytotoxic. The LPO content in the stressed group was significantly higher than that in the control group (P < 0.05; Table 5). We measured hepatic TNF-α levels in both control and stressed groups. As shown in Table 5, stress markedly increased hepatic TNF-α levels. Apoptosis-associated proteins caspase 9, cytochrome c and caspase 3 were significantly elevated in the stressed group (38.45 ± 1.39, 232.50 ± 3.54, 81.45 ± 3.23) compared with those in control group (34.34 ± 0.43, 187.22 ± 5.36, 68.28 ± 4.06; P < 0.05).

**Table 5:** **Levels of lipid peroxides and apoptosis-associated proteins**

	Control	Stress
**LPO (nmol/mg)**	0.32 ± 0.03	0.44 ± 0.09^a^
**TNFα (ng/L)**	4800.00 ± 494.97	7075 ± 106.07^a^
**Caspase 9 (IU/L)**	34.34 ± 0.43	38.45 ± 1.39^a^
**Cytochrome c (nmol/L)**	187.22 ± 5.36	232.50 ± 3.54^a^
**Caspase 3 (IU/L)**	68.28 ± 4.06	81.45 ± 3.23^a^

^a^the value in the stressed group is significantly different from that in the control group, P < 0.05.

**Figure 7:**
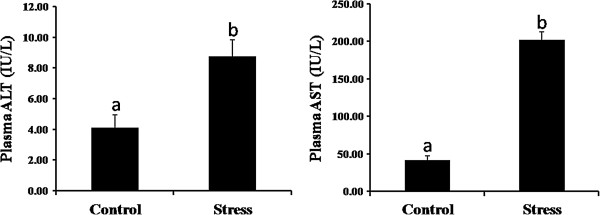
**Liver injury induced by stress in** ***Coilia nasus*** **(** ***n*** **= 15).** Plasma samples were collected 0.5 h after challenge, and plasma transaminase (ALT and AST) concentrations were determined. Bars represent the mean for each replicate or treatment ± SEM. Replicates or treatments with different letters are significantly different at P < 0.05.

Plasma aminotransferases were assayed to evaluate hepatotoxicity in *Coilia nasusu*. The ALT and AST concentrations in the control group were 4.10 ± 0.85 and 41.45 ± 7.79 IU/L, while in the stressed group the concentrations were 8.76 ± 1.08 and 202.12 ± 10.01 IU/L, respectively. The results indicate that significantly increasing plasma levels of ALT and AST developed following stress (Figure 7).

## Discussion

The physiological and biochemical response to exposure to stressors has been studied widely in teleosts. One of the evolutionarily conserved stress responses is the rapid elevation in plasma cortisol and plasma glucose levels in response to stressor challenges [22]. In teleosts, the production of cortisol is under the control of the hypothalamus–pituitary–adrenal (HPA) axis [23, 24]. Cortisol is released from the adrenal tissue, resulting in up-regulation of the plasma glucose level. Therefore, plasma cortisol and plasma glucose can be used as indicators of the stress response. In our experiment, these parameters were significantly elevated in the stressed fish when compared with the control fish (Figure 1), this suggested that our sample could be used for transcriptome sequencing.

We found that 2,990 genes were significantly up-regulated, while 3,416 genes were significantly down-regulated in response to loading stress (Figure 5). On pathway enrichment analysis, “metabolism” and “immunity” were the two most frequently represented categories. In the “metabolism” category, “carbohydrate metabolism” and “lipid metabolism” were major subclasses, and included the “glycolysis/gluconeogenesis”, “starch and sucrose metabolism”, “glycerophospholipid metabolism” and “fat digestion and absorption” pathways. These carbohydrate metabolic pathways are mainly involved in blood glucose elevation (gluconeogenesis) and glucose utilization (glycolysis). The transcriptional expression of the rate-limiting enzymes (glucose-6-phosphatase and glucokinase) in this process was detected by RT-qPCR, both enzymes were significantly increased following stress (Figure 6). In addition, plasma glucose levels were significantly elevated after stress (Figure 1B), which provided further confirmation of the findings. These results are in agreement with studies that have reported higher activities of glycolytic enzymes after exposure to an acute stressor in fish, this may be necessary to cope with the increased energy demand of the liver, including enhanced gluconeogenesis, that is required to re-establish homeostasis [24]. Those two lipid metabolism pathways are involved mainly in fat digestion, absorption and oxidation. Transcript levels for the rate-limiting enzymes (hormone-sensitive lipase and carnitine actyltransferase I) in adipokinetic activity and the fatty acid beta-oxidation process were also significantly increased after stress.

Taken together, these results suggest that molecular regulation of enzymes critical for energy substrate mobilization and utilization is a key mechanism involved in acute stress adaptation in fish. However, problems may be associated with these processes, for example, the fatty acid beta-oxidation process may produce lipid peroxides (LPO) [25]. High levels of LPO or plasma glucose may induce an increase in TNF-α [26]. It is now well accepted that trimerization of the respective receptor by TNF leads to the assembly of the death-initiating signaling complex (disc) [27]. The disc consists of the death domain of the receptor, one or several associated proteins, and procaspase-8. Upon assembly of the disc, procaspase-8 is autocatalytically cleaved and activated [27]. The active caspase-8 can either process downstream effector caspases such as caspase-3, -6, and -7 directly [28, 29] or activate mitochondria as an amplification mechanism [30]. If mitochondria are involved, caspase-8 cleaves bid, a protein of the Bcl-2 family [31]. The C-terminal portion of the bid molecule inserts into the outer mitochondrial membrane and induces the release of cytochrome c into the cytosol [31, 32]. Cytochrome c, together with dATP, apoptosis activating factor-1 (Apaf-1) and procaspase-9, forms the apoptosome, which leads to the activation of caspase-9 and subsequently caspase-3 [33]. Caspase-3 and other effector caspases cleave death substrates, leading to apoptosis [34]. In our study, the levels of TNF-α, caspase 9, cytochrome c and caspase 3 in the liver were significantly elevated in the stressed fish compared with the control fish (Table 5). ALT and AST are important indicators of liver injury, and were also significantly elevated (Figure 7). These lines of evidence suggested that loading stress induces liver cell apoptosis mediated by TNF-α, which causes liver injury.

## Conclusions

In the present study, using Illumina sequencing and bioinformatics analysis we analyzed the liver transcriptome of *Coilia nasus* that were stressed by loading. The main objective of this study was to annotate genes from this transcriptome analysis and identify potential stress gene and signaling pathways. On the basis of the transcriptome date, we explored the mechanism by which loading stress causes death in *Coilia nasus*. A total of 65,129 unigenes were generated, the mean unigene size and N50 were 607 bp and 813 bp, respectively. Of the assembled unigenes, we identified 2,990 genes that were significantly up-regulated, while 3,416 genes were significantly down-regulated in response to loading stress. Pathway enrichment analysis based on loading stress-responsive unigenes identified significantly stress-related pathways. “Metabolism” and “immunity” were the two most frequently represented categories. In the “metabolism” category, “glucose metabolism” and “lipid metabolism” were the major subclasses. The transcriptional expression of rate-limiting enzymes in “glucose metabolism” and “lipid metabolism” was investigated by RT-qPCR, all were significantly increased following stress. Apoptosis-associated proteins TNF-α, caspase 9, cytochrome c and caspase 3 were significantly elevated in the stressed group, indicators of liver injury (ALT and AST) were also significantly elevated, which indicates that loading stress induces liver injury in this fish.

### Availability of supporting data

Raw sequencing data is available through the NCBI Sequence Read Archive under Project Accession SRP034828 (http://www.ncbi.nlm.nih.gov/). All samples were sequenced as 90 bp paired-end reads on an Illumina HiSeq 2000 sequencer.

## Electronic supplementary material

Additional file 1: Table S1: Genes and specific primers used for real-time PCR. (DOCX 12 KB)

Additional file 2: Table S2: KEGG pathway analysis for all unigenes. (XLSX 21 MB)

Additional file 3: Table S3: List of the genes up-regulated or down-regulated in response to stress. (XLSX 2 MB)

## Abbreviations

RNA-Seq: RNA sequencing
GO: Gene ontology
RPKM: Reads/Kb/Million
ALT: Alanine aminotransferase
AST: Aspartate transaminase
TNF-α: Tumor necrosis factor alpha
Cyt c: Cytochrome c.
