# Supplementary material for: Transcriptome analysis gene expression in the liver of Coilia nasus during the stress response
Source: BMC Genomics. 2014 Jul 4;15(1):558. doi: 10.1186/1471-2164-15-558 (PMC4094779; doi:10.1186/1471-2164-15-558)
Supplement: Supplementary file 1 — Additional file 1: Table S1: Genes and specific primers used for real-time PCR. (DOCX 12 KB) [file 12864_2013_6232_MOESM1_ESM.docx]

| **Primer** | **Sequence** | **Gene** |
| --- | --- | --- |
| 31 | GACCTCGGCGGCACCAAC | Glucokinase |
|  | CCTGTCATGGCGTCCTGTGG |  |
| 32 | CATATGGGTCGCTGTGATTGG | Glucose-6-phosphatase |
|  | GGTTGGTCTGGTGTCGGCAC |  |
| 34 | TGGCGCAACTCATCCTGGT | Carnitine actyltransferase I |
|  | ATCCGTTTGACTGTTGCACCAT |  |
| 35 | GGACGATTCGGTGATGTTTGC | Hormone-sensitive lipase |
|  | TGTTGAAAGACTTCTCTTATTCGCTG |  |
| B | AACGGATCCGGTATGTGCAAAGC' | Beta actin |
|  | GGGTCAGGATACCTCTCTTGCTCTG |  |

Table S1. Genes and specific primers used for real-time PCR
